# Synthetic biology regulation in Europe: containment, release and beyond

**DOI:** 10.1093/synbio/ysad009

**Published:** 2023-04-20

**Authors:** Lalitha S Sundaram, James W Ajioka, Jennifer C Molloy

**Affiliations:** Centre for the Study of Existential Risk, University of Cambridge, Cambridge, UK; Department of Pathology, University of Cambridge, Cambridge, UK; Colorifix Ltd, Cambridge, UK; Department of Chemical Engineering and Biotechnology, University of Cambridge, Cambridge, UK

**Keywords:** regulation, EU, synthetic biology, biotechnology, containment

## Abstract

While synthetic biology is hoped to hold promise and potential to address pressing global challenges, the issue of regulation is an under-appreciated challenge. Particularly in Europe, the regulatory frameworks involved are rooted in historical concepts based on containment and release. Through a series of case studies including a field-use biosensor intended to detect arsenic in well water in Nepal and Bangladesh, and insects engineered for sterility, we explore the implications that this regulatory and conceptual divide has had on the deployment of synthetic biology projects in different national contexts. We then consider some of the broader impacts that regulation can have on the development of synthetic biology as a field, not only in Europe but also globally, with a particular emphasis on low- and middle-income countries. We propose that future regulatory adaptability would be increased by moving away from a containment and release dichotomy and toward a more comprehensive assessment that accounts for the possibility of varying degrees of ‘contained release’.

**Graphical Abstract** 
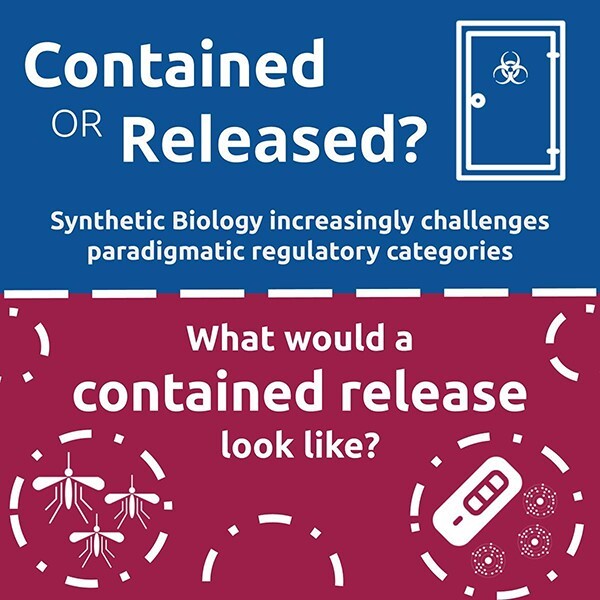

## 1. Introduction

Synthetic biology and other engineering biotechnologies have emerged as potentially transformative to industries across the world. According to the Organisation for Economic Co-operation and Development, at least fifty countries have a national bioeconomy strategy (1), many of which refer directly or indirectly to synthetic biology as a potential driver of innovation. Although numerous technical challenges remain if synthetic biology is to deliver on all its promises (2), policy and societal factors are equally important, as these will be the ultimate checkpoint that determines market success and widespread adoption. Key among these factors is regulation: the legal means by which the technical work is performed and eventually developed into a product within the bioeconomy. The regulation of synthetic biology is therefore an area that merits particular consideration in light of achieving the transformative outcomes anticipated across areas as broad as energy, agriculture, environment, manufacturing and health.

The risk of regulation not keeping up with technical developments is a long-acknowledged one (3). As noted in the UK government’s 2016 Strategic Plan on Biodesign for the Bioeconomy, ‘Regulations will need to be framed to accommodate continuously evolving frontiers of knowledge and understanding …’ (4). Yet the foundational concepts of genetically-modified organism (GMO) process-focused regulations were developed decades ago when genetic engineering was technically feasible but remained costly and slow.

This is a concern in terms of both increasing volume and widening scope of potential synthetic biology products. Many regulatory regimes are based on case-by-case assessments. The evolution of regulation is shaped and constrained by previous adaptations to now historical environments and technological landscapes, which place limitations on its agility. Increasing numbers of products will arrive that fall between categories or weaken existing standard regulatory frameworks and risk assessment approaches. For example, the ability to synthesize ever-larger segments of genetic material at ever-lower costs weakens the concept of a ‘reference organism’ that is at the heart of many comparative regulatory frameworks which compare engineered organisms to their ‘parents’. In principle, entire chromosomes or even genomes can be engineered rather than the limited number of genes possible by traditional cloning methods. This is even more stark in the area of xenobiology, where the genetic rules themselves are non-canonical with expanded sets of nucleotides and amino acids producing organisms that cannot exchange functional genetic material with ‘wild’ non-GM strains.

Finally, the imaginaries promised by synthetic biologists are rooted in moving from familiar processes of industrial biotechnology within designated facilities to applications where engineered organisms themselves are used outside the laboratory. European GM regulations were developed with a primary legislative categorization of GM use being ‘contained use’’ within defined premises and ‘deliberate release’ into the environment. This distinction dominates assessments to date and yet so narrowly defines the boundaries of contained use that it fails to account for products which are ready to deploy today. It is likely to continue to fail for a large number of pipeline products at high technology readiness levels.

In this paper, we use examples from our own experience working on projects that draw out some of the specific ways in which the contained use and deliberate release categorization falls short. Colorifix, the first case study illustrates the process of using non-traditional fermentation facilities for nevertheless ‘contained’ uses and derives from a start-up co-founded by J.W.A. The arsenic biosensor tackles the issue of a ‘contained release’ where a product is genetically and physically contained but designed for use in a field setting in a low- or middle-income country (LMIC). Both J.W.A. and L.S.S. worked for several years on this project. As part of this project, we also researched alternative models of regulation that might be applicable, including ‘mobile deployment’ as in our third case study. Our fourth case study explores the application of deliberate release regulations to GM insects developed by the UK company Oxitec, with whom J.C.M. performed doctoral research, and our fifth examines the emerging case of cell-free synthetic biology, which currently falls outside the scope of GM regulation. All three authors have considered the regulatory status of cell-free systems in the context of diagnostics and biosensors. These case studies dissect the specific implications of the somewhat artificial split of ‘contained use’ and ‘deliberate release’ on concrete synthetic biology projects. We then explore some of the possible consequences of these shortcomings, not just on the individual projects themselves, but how these consequences might manifest as signals to the field of synthetic biology as a whole, and in the realm of international development and the establishment of healthy local bioeconomies in LMICs.

## 2. Historical background to Genetically-Modified Organism regulation in the European Union

It is generally acknowledged that the European Union (EU)’s approach to regulating genetic modification is based on the precautionary principle as applied to ‘process’. (The precautionary principle as applied by the EU is first mentioned in Article 191 of the Treaty on the Functioning of the EU which places it as a key concept in European environmental law. However, neither this nor future European law provides a strict definition of the principle or its consequences. The closest is from the Communication on the precautionary principle from 2000: ‘Recourse to the precautionary principle presupposes that potentially dangerous effects deriving from a phenomenon, product or process have been identified, and that scientific evaluation does not allow the risk to be determined with sufficient certainty. The implementation of an approach based on the precautionary principle should start with a scientific evaluation, as complete as possible, and where possible, identifying at each stage the degree of scientific uncertainty’ (5)). This emphasis on process therefore means that it matters very much what method has been used to produce an organism and this acts as the ‘trigger’ for regulation. It also matters a great deal, legally speaking, where the products are physically deployed: this is what differentiates ‘contained use’ from ‘deliberate release’.

These lines were plainly drawn as early as 1986, in a ‘Communication from the Commission to the Council: A Community Framework for the Regulation of Biotechnology’(6). Here, a clear distinction was made between genetically modified organisms in ‘enclosed manufacturing systems, and the products produced by such methods’ and those in agricultural and environmental settings, where a debate was already growing about GM crops. The Communication largely concluded that existing laboratory regulation was sufficient, that ‘planned release’ was still too early in its development to regulate explicitly and that work would be undertaken to flesh out some guidelines). These formally set the frame by which synthetic biology continues to be regulated in Europe today and the distinction has persisted through numerous amendments. This split is clear from some of the first pieces of relevant EU law-making: Directive 90/219/EEC on contained use (7) and Directive 90/220/EEC on deliberate release (8).

The goals of the legislation were safety, risk mitigation and harmonization between Member States. For ‘contained use’ applications, a clear line is drawn between Type A, which we might call ‘academic’ uses of GMOs (‘teaching, research, development, or non-industrial or non-commercial purposes and which is of a small scale (e.g. 10-l culture volume or less)’, and Type B, essentially any other scale or purpose. The requirements are slightly different in terms of reporting and notification, but, throughout the Directive, it is clear that containment is the key means by which safety and risk mitigation are to be achieved, with the main duties to be undertaken pertaining to notification, the application of ‘good microbiological practice’ and accident reporting. This Directive allows Member States some degree of leeway in the implementation of their own national guidelines, as long as the minimal qualitative criteria set forth in 90/219/EEC were met (9).

The ‘Deliberate Release’ Directive 90/220/EEC did not allow for this, instead applying directly to all Member States. It emphasized a ‘step-by-step’ approach, with each stage involving a gradual reduction in containment and concomitant increase in scale. When placing on the market is eventually sought, this is to be a ‘Community authorization’ meaning that the application must be filed in one Member State but if it recommends approval, then all EU members are allowed to lodge objections to be resolved by voting if needed.

Importantly, for the designations under which products are to be placed on the market, the Directive ‘should not apply to products containing, or consisting of, GMOs covered by other Community legislation’. That is to say, as we shall see later, food and feed, and animal and human medicines would be subject to other Directives. In terms of definitions, the salient one that carries forth into subsequent legislation and literature is that of a ‘deliberate release’ itself, being ‘any *intentional* introduction into the environment of a GMO or a combination of GMOs *without provisions for containment* such as physical barriers or a combination of physical barriers together with chemical and/or biological barriers used to limit their contact with the general population and the environment’ (emphases added).

In effect, the embodiment of European GMO legislation in these two separate documents itself reflects a staged process, with GMOs being studied and experimented with in a relatively ‘low stakes’ laboratory environment, then proceeding to the stage-gated phase of deliberate release to the environment (10).

Taken together, these two pieces of European legislation have, since at least 1990, had two main effects: they codified the line of demarcation between contained use and deliberate release and established the precautionary principle as the underpinning strategy for decision-making.

The current iteration of the Deliberate Release Directive was introduced with 2001/18/EC (repealing 90/220/EEC) (11). Directive 2001/18/EC served a number of purposes, clarifying the procedures to be undertaken in the ‘step-by-step’ process from the earlier directive. The new Directive also emphasized the need for labeling, traceability and the keeping of registers. It can be said that the new Directive also fulfilled political and trade-related purposes, ending what had been a *de facto* ban on the import and placing on the market GM products by, for example, Monsanto (decided through a European Court of Justice ruling) or Canada, the USA and Argentina (in World Trade Organization cases) (10). In addition to 2001/18/EC, other Directives also impact products involving genetically modified organisms destined for market in a sectoral manner. These regulations typically involve the use of GMOs in food or feed (12) or for the purposes of animal or human medicinal products (13).

In the same intervening years since 1990, a number of amendments and related Directives and decisions were passed that had a bearing on contained use, including the implementation of classification systems and detailed procedures for the examination of GM microorganisms in terms of safety for human health and the environment. These were consolidated and updated in a 2009 Directive, 2009/41/EC (14). Key to the Directive’s implementation is its definition of ‘Containment and other protective measures’ which in effect requires the microorganism to be used in a laboratory environment. For Containment Level 1, the lowest level that is typically applied for organisms that have minimal risk to health or the environment, the requirements are a work surface that is resistant to water, acids, alkalis, solvents, disinfectants and decontamination agents, and easy to clean; an autoclave onsite and suitable protective clothing in addition to other general principles that presuppose work will happen in a laboratory.

Alongside these clarifications, the Directive also purports to institute ‘Increased flexibility for amendment of the technical annexes, allowing timely adaptation to scientific and technical progress’ (15), as demonstrated by the Preamble to the Directive, which states: ‘In particular, the Commission should be empowered to adopt the amendments necessary to adapt Annexes II, III, IV and V to technical progress, and to adapt Annex II, Part C.’

The Annexes are as follows:

‘I: lists (non-exhaustive) of whether particular microbiological techniques result in genetic modification;

IIA: list of techniques that could result in microorganisms excluded from the Directive’s purview;

IIB: a list of criteria to be fulfilled by microorganisms, in terms of their safety for human health and the environment, before their inclusion into Part C;

IIC: a list of genetically modified microorganisms that, having met the criteria in the above Part B of Annex II, are thus excluded from the scope of the Directive;

III: procedures for assessment and subsequent classification;

IV: the ‘normal’ minimum requirements for containment (presumed here to be a workplace or laboratory) and

V: notification procedures.’(14)

Part C of Annex II of this Directive is therefore designed to include a list of organisms that, fulfilling certain general and specific criteria of safety for human health and the environment, might be excluded from the scope of the Directive as a whole, if those organisms are used under conditions of containment. That is to say, after assessment for safety to human health and the environment, that organism could then be exempt from the Directive, through placement on the list in Part C of Annex II. The list is, at the time of writing, empty and the implications of this are clearly seen in our second case study.

Thus, it would seem that the several aspects of the regulatory procedure regarding contained use—as delineated in Directive 2009/41/EC—should be adaptable to take into account scientific advances, including specific techniques and containment measures. Most notably, they appear to provide a mechanism for microorganisms that are intended to be used under conditions of containment and are shown to be safe for human health and the environment to be excluded from the full scope of the Directive by listing them in Annex II, Part C.

It is against this backdrop of European legislation that we consider four case studies of products that have attempted to go through regulatory processes and hit barriers due to historical assumptions that GMOs will either be in a fully equipped lab or openly in the environment. We also discuss the implications of new forms of genetic containment and synthetic biology platforms that in various ways do not conform to the expectations of the existing legislation. We suggest that in order to adapt to advancing genetic technologies, contained use and deliberate release may require a spectrum rather than the binary definitions that underpin current regulations.

## 3. The limits of laboratory containment: retrofitting biomanufacturing in textile dye houses

The global textile industry is valued at nearly one trillion dollars annually (16) and is responsible for upwards of 20% of industrial wastewater pollution. Textile wastewater includes over seventy highly toxic chemicals, at least thirty of which cannot be retrieved (17, 18). One approach to avoid the use of toxic chemicals, taken by Colorifix—a UK-based synthetic biology company co-founded by J.W.A.—has been to engineer microorganisms to perform the dyeing instead, taking advantage of the natural ability of bacteria to stain organic materials.

Colorifix has specific regulatory needs because (i) it uses recombinant microbial fermentation liquor to directly dye textiles, rather than purifying the pigment products; (ii) in order to scale, this process needs to occur ‘within’ dyeing facilities through retrofitting to existing plant and not in a custom built fermentation facility.

The process is technically GMO Contained Use as defined in the EU, but the procedure is sufficiently different from standard GM biotechnology that the route for licensing was unknown. Dye houses do not look like typical fermentation facilities and do not have a history of using GMOs or genetically-modified micro-organisms (GMMs). Colorifix identified the appropriate regulatory body or bodies to approach in each client’s country and then provided them with the documentation and evidence of competence to grant a license. In Portugal, an EU country, obtaining the license was straightforward with an application through one agency. The process took >8 months for the first license, but only ∼1 month for the second and third licenses. In India, three regulatory agencies required a petition, an application, a review and an interview with the GMO council. The license was eventually granted but with the condition of forming a biological safety committee on site. In Bosnia, a non-EU country, the use and import of GMOs were banned prior to Colorifix engaging with a dyehouse for a license. The process is now nearing completion after > 3 years of meetings and discussions with government ministries, including a change in legal status for working with GMOs.

This case study illustrates that GMMs emerging from synthetic biology companies are already being deployed in new industrial environments and that the current flexibility of contained use regulations within the scope of defined premises can be sufficient to enable this. The containment does not have to take place in a lab, but the premises must have evidence of facilities that conform to expectations. In some countries such as India, governance and oversight are expected to echo that of more traditional biotech facilities but in the case of Colorifix, to date regulation has not blocked deployment, even if it has added transaction costs and delays.

## 4. ‘Contained release’: an arsenic biosensor as a GM product falling between the two regulatory paradigms

**Figure 1.**
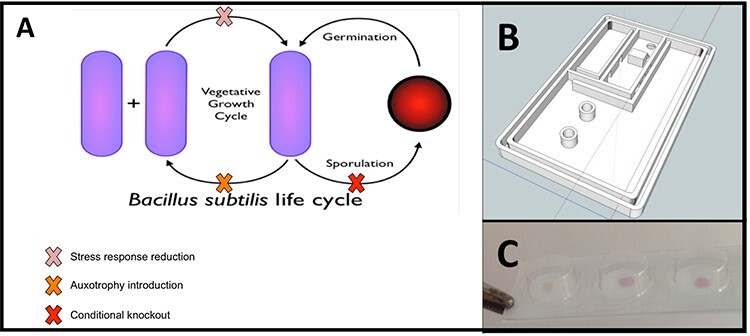
Triple forms of genetic containment (A) and two forms of physical containment in a plastic case (B) and blisters (C) in the Arsenic Biosensor Collaboration.

Significantly more challenges may be encountered once the use of a GM product moves beyond defined premises and into the outside world. The Arsenic Biosensor Collaboration is an example of a synthetic biology project that challenged regulatory categorization in the EU. The project, which ran from 2012 to early 2016, was led by J.W.A. with L.S.S. as a member of the research team. It sought to develop a whole-cell biosensor to detect arsenic at different threshold levels in well water in Nepal. The planned biosensor consisted of several physical and genetic components (Figure 1) designed to make the engineered organism as safe as possible while also satisfying the operational requirements of a field-use biosensor.

*Bacillus subtilis* was used as the ‘chassis’ (the organism into which exogenous genes encoding the sensing, amplification and reporting machinery would be inserted). This is a well-characterized, nonpathogenic organism long used in laboratories. Moreover, *B. subtilis* is frequently used in industrial processes and fermented foodstuff (19, 20), and products made in this organism have been granted the generally regarded as safe status by the U.S. Food and Drug Administration (21). The Arsenic Biosensor strains additionally introduced disabling mutations that made it ‘less fit’ than its parental form and unable to persist in the environment. These included disruptions to the competence and oxidative stress response pathways, as well as auxotrophies and conditional sporulation.

The bacteria were further contained within the device by spotting onto filter paper with pores less than the size of *B. subtilis* covered by a polycarbonate blister that was optically welded to the filter paper to prevent any leakage. Blister packs were contained within a hard polycarbonate packaging that was designed to be filled with water (to be tested for arsenic) and then self-seal. Not only the engineered bacteria but also any water that had come into contact with them would be sealed inside the container. The polycarbonate shell’s melting temperature was higher than the inactivation temperature of *B. subtilis*, thus minimizing release risk even if the plastic was compromised by exposure to heat. Each of these innovations can be seen as examples of safety-by-design: a process by which ‘through technical or procedural interventions, potential technology hazards are removed early in the development process and evaluated iteratively throughout development’ (22).

Although the device was designed for use in Nepal in the first instance, J.W.A., L.S.S. and other members of the research team were keen to obtain local (UK) approval, either in parallel to or before field use in Nepal. This was primarily for ethical reasons, as the practice of researchers from high-income countries testing unregulated technologies in LMICs is problematic and has a long history of misuse resulting in harm to local communities (23), a practice that the European Commission calls ‘ethics dumping’ (24, 25). Local stakeholder engagement reinforced this view: regulatory approval in the UK would significantly ease the process in Nepal, as obtaining direct approval from the regulatory authorities in Nepal also did not prove fruitful as there was no apparent regulatory framework equipped for the purpose.

In the UK (then an EU member), the regulation of genetically modified organisms is categorized as either ‘contained use’ or ‘deliberate release’. Meetings were held with the national competent authorities: the Health and Safety Executive (HSE) for contained use and the Department for Environment, Food & Rural Affairs (Defra) for deliberate release. Given the many measures taken to ensure containment described earlier, it seemed inappropriate to those present at the meetings for the biosensor to be regulated as a ‘deliberate’ release. In the text of the relevant Directive, EC/2001/18/EC, the definition of ‘deliberate release’ is after all ‘the intentional introduction into the environment of a GMO or a combination of GMOs *for which no specific containment measures* are used…’ (emphasis added) (11). The project’s intent was not to introduce the GMO into the environment; several specific containment measures were taken to prevent this. We call this scenario a ‘contained release’.

Upon advice from the national competent authorities, the research team then considered the Contained Use Directive (2009/41/EC) in more detail. Specifically, and in keeping with that Directive’s stated desire to be adaptable to technical progress (‘In particular, the Commission should be empowered to adopt the amendments necessary to adapt Annexes II, III, IV and V to technical progress, and to adapt Annex II, Part C’), it was thought that Parts B and C of Annex II might apply and thus exempt the GMM from the Directive.

To apply for the biosensor bacterial strains’ inclusion in this list, the team prepared a dossier for consideration at a national level by the UK’s Scientific Advisory Committee on Genetic Modification in 2013. This body was satisfied with the triple containment (genetic and double physical) of the organisms, and the application was passed to the European Comissions (EC)’s Directorate General for Health and Consumer Protection of the European Commission (DG-SANCO) by the HSE (26). After 8 months of attempts to contact DG-SANCO, the applicants were told that the application needed to be resubmitted.

Following a later meeting of HSE and Defra with representatives from DG-SANCO, additional guidance was given, in the form of Guidance Document (2005/174/EC) on ‘establishing guidance notes supplementing part B of Annex II to Council Directive 90/219/EEC on the contained use of genetically modified micro-organisms’ (27). Neither 90/219/EEC nor its Guidance Document was in force at the time of the request. Nevertheless, a revised application based on this older Guidance Document was submitted. It was then decided that, although the biosensor organism was intended neither as food nor feed, it was to be reviewed by the European Food Safety Authority (EFSA), as this body had a standing GM review committee (28). Six months later, the EFSA convened a review and examined the dossiers.

The result was a request for further information (29). However, as the process was a non-binding consultation by the EFSA on behalf of DG-SANCO, it was not known whether receipt and positive assessment of this further information by the EFSA would be enough to place the biosensor organism in Annex II, Part C, nor whether all Member States would have to approve this nor what the alternatives were in order to move forward. No specific body was designated as having the authority either to approve or reject the application or to provide guidance. The feeling among the researchers was that, while the project was informally described as beneficial and worthwhile, nobody was comfortable to take responsibility for approval.

While waiting for the deliberations to occur, grant funding for the project expired and, given the degree of uncertainty regarding eventual approvals, was not renewed. As a consequence, the research team dispersed and work on the project proceeded toward a ‘cell-free’ or *in vitro* mode of operation which would workaround the need for a ‘contained release’ of organisms by avoiding using living organisms.

While there was theoretically an EU procedure to adapt regulation for a contained release by listing a GMO on Annex II, Part C, this was a path that had never been taken. The impacts here are 3-fold. First, lack of clarity about the process to achieve regulatory approval introduced too much uncertainty for existing and future funders to retain confidence in the near-term impact of the technology. This uncertainty affected the research group’s ability to plan ahead and retain key staff, as well as to maintain relationships with stakeholders in Nepal and later in Bangladesh, where interest in the biosensor had also been high. The Annex II, Part C regulatory pathway may have eventually borne fruit, but the extreme uncertainty made it impossible to continue the project.

Second, researchers in the synthetic biology community—having followed this case with interest—might conclude that this type of project, ‘with this type of regulatory approval’, is simply unfeasible and concentrate instead on applying their skills and imagination to more traditional, ‘contained’ work. Other groups may decide to pursue different biological strategies not because they are necessarily better at fulfilling the specifications of the project or because they are biologically safer, but because they are more regulatable in the short term. Third, the lack of a named body with the authority to regulate products intended for contained release is especially problematic if synthetic biology is to be the transformative industry many hope it will be.

The Arsenic Biosensor Collaboration is emblematic of the difficult journey that even relatively uncontroversial synthetic biology products might face. It also demonstrates how easily a lack of clear regulatability can collide with the vagaries of funding cycles to render a project ultimately unviable.

## 5. The limits of laboratory containment: mobile deployment

A less radical containment strategy was employed by another team in Germany seeking to develop a field-use arsenic biosensor for eventual deployment in Bangladesh: ARSOlux. This biosensor consists of *E. coli* engineered with bacterial luciferase under the ArsR promoter so as to emit light proportionate to arsenic concentration (30). According to the team, ‘obtaining support and approval from the Bangladeshi government for the commercial deployment of the device has proved to be a more difficult problem. The lack of a clear regulatory framework for GMOs and the political instability in the country have further exacerbated the challenges faced by the ARSOlux team’ (31).

In Germany, the national regulations follow the same structure and classifications as the EU Directive but are administered at the state level. The ARSOlux team submitted a notification of contained use to the Saxon competent authority (Regional Office for Environmental Protection Saxony-Anhalt—Laboratory for the Monitoring of Genetic Engineering, *Gentechnisches Überwachungslabor am Landesamt für Umweltschutz Sachsen-Anhalt*), but rather than use the provision under Annex II Part C, the team instead adapted their ‘containment’ such that they could proceed under the existing standards of containment documented in the Directive. They equipped a van to act as a mobile laboratory to which the provisions of personal protective equipment, waste disposal, accident recording and other requirements would apply. The use of the biosensor within such a van was approved (30), with the arsenic tests to be performed by trained scientists. These requirements fall under 2009/41/EC, and so the application did not need to be submitted to the EC.

Contained use in a van was only reviewed and approved in Saxony, but this has been enough to foster confidence in other countries that may lack their own regulatory frameworks for this type of product to proceed with development. While the need for a specially equipped van and trained scientists precludes the sale of the biosensor as an ‘off-the-shelf’ test for use by individual consumers in low-resource settings, the van and biosensor may well be considered as sufficient ‘containment’ by other countries as well. As such, by employing a ‘portable containment’, it may be that the regulations themselves are in a way rendered portable. This case study supports the finding by Colorifix that so long as there is an identifiable facility, there is flexibility within the contained use regulation to accommodate a number of non-laboratory environments.

## 6. Containment measures for the deliberate release of non-plant organisms

It may well seem that it would have been simpler for those teams developing an arsenic biosensor to go through the Deliberate Release Directive, which has been in place since 2009. This Directive, however, is mainly geared toward plants and crops based on the historical context of the legislation. Products falling under this Directive are split into two phases: Phase B for trials (‘experimental releases’) and Phase C for placing on the market. At present, 1601 projects have been awarded trial permits. The majority (915) are plants, and all but one of the 686 non-plant permits involve clinical trials of medicinal products. (There is mention of a research biosensor from KWR Research, but there is no further trace in the literature of this product following the award of the trial permit).

The trial phase of deliberate release follows a ‘Step-by-step’ principle of ‘progressively decreasing physical containment’ to allow ‘a logical, incremental step-wise process whereby safety and performance data are collected’ (32). While there is no strict definition of a ‘step’, a 1998 appraisal for the European Parliament of the working in practice of Directive 90/220/EEC on the deliberate release of genetically modified organisms (33) states that initially it was believed that every single case would follow a sequence of testing in greenhouses, small-scale trials and large-scale trials before ending in a market application.

Here, we identify case studies where synthetic biology is giving rise to technologies that challenge these assumptions in various ways.

### 6.1 Genetically modified insects

The purpose of the deliberate release regulations is to ensure that, albeit deliberate, releases are nevertheless constrained. The purpose of releasing GM crops is to have them grow but not spread; much effort is spent in proving that GM and non-GM crops of the same species will not undergo cross-pollination, which is achieved through the physical separation of crops and genetic containment strategies such as sterility. Expectations are different when it comes to modified insects which are released for population control. In this case, the interaction with wild-type organisms and the subsequent effects on the ecosystem are the aim, rather than an undesirable consequence.

A mismatch in assumptions and understanding of the purpose of the technology therefore results in regulations being applied beyond the originally intended applications, because there is no alternative. This is in contrast to a case like the Arsenic Biosensor Collaboration where the chosen regulatory path, had it unfolded predictably, would have been the appropriate one.

The only applications for GM insect trial releases in the EU have been Oxitec’s OX3097D olive flies proposed for a field trial in Spain. OX3097D flies contain a transgenic construct that results in the mortality of offspring from matings between OX3097D flies and wild-type flies and is therefore inherently ‘self-limiting’ because the transgenes are lethal to all organisms carrying them through early development (34). Continuous introduction of the gene is required through the release of additional flies that are reared under conditions where the lethal phenotype is not activated. This application was withdrawn after a lengthy exchange with the Spanish authorities. The company was asked to restructure the confinement measures of their planned field trials but, 3 years later, this was still not deemed sufficient with Oxitec stating: ‘We cannot even get to the first hurdle of getting a genetically modified insect in a field cage’ (35). As with the Arsenic Biosensor Collaboration case study, the issue appears to have been a reluctance from authorities to outright refuse permission, but the process ended up being sufficiently convoluted and drawn out as to result in the abandonment of the project—or at least of that particular regulatory strategy.

In both cases, the end point of pursuing a particular regulatory path was never an outright refusal of permission. Instead, the process can almost be considered one of attrition, where the lack of a visible finish line combined with a lack of transparency and clarity in the reasoning behind the regulatory process rendered it ultimately unviable.

### 6.2 Containment and release without organisms: regulation and cell-free synthetic biology

Both contained use and deliberate release regulation are targeted at the containment of GMOs and GMM (organisms and microorganisms, respectively). *In vitro* manipulation of DNA has historically fallen outside the scope of GM regulations, but its increasing sophistication has started to raise questions about containment.

Increasingly, work in synthetic biology has moved toward using cell-free systems. Here, genetic circuits are designed and constructed within cells in a laboratory under the usual containment levels and processes. The desired functionality is achieved, and these modified cells are lysed, releasing the cellular machinery. Cell-free systems have numerous applications, such as rapid prototyping of genetic circuits including biosensor designs, metabolic engineering, industrial biotechnology, application of unnatural amino acids and more.

The regulatory status of cell-free products for use outside containment remains unclear. The absence of a cell has, at least by some researchers, been seen as ‘a promising mechanism for circumventing GMO release’ (36) or indeed ‘many of the biosafety and biocontainment regulations that exist for living cells’ (37). Recently, several diagnostic products lysing cell-free synthetic biology have been developed (38–40), and the question of regulation stemming from the cell-free nature of these products has not been raised as a particular concern (although they would be subject to separate medical device regulations if commercialized).

The assumption, therefore, is that regulation of a genetically modified organism is moot in the absence of an organism (interpreted as a living cell). In EU Directives pertaining to GMOs, however, an ‘“organism” means any biological entity capable of replication or of transferring genetic material’ (11) in the Deliberate Release Directive, extended to ‘any microbiological entity, cellular or non-cellular’ in the Contained Use Directive’s definition of ‘micro-organism’ (14).

Whether cell-free systems would therefore be covered is a matter for regulatory lawyers to decide but the situation certainly is not as simple as ‘no cell = no regulation’. There is uncertainty about whether the ‘biological definition’ and the ‘legal definition’ will correspond and who will end up making that judgment. To date, no cell-free organism has been produced or brought to market that deals with non-diagnostic uses, in which case the GM regulations could be the only relevant regulatory tool. (There may be standards to do with environmental sensors, but these are not the same.) Until there is a ‘first mover’ (as in the case of the Arsenic Biosensor Collaboration) who seeks regulatory approval for a non-medical and non-veterinary use of a cell-free synthetic biology product, this is unlikely to be resolved with any clarity.

These definitions are particularly important given the ongoing development of synthetic cells, whereby cell-free extract is encapsulated in a synthetic membrane and is capable of producing proteins and other genetic material (41). Work is underway to produce versions that are capable of self-replicating, at which point it is likely they will fall within the scope of the regulation. It has already been demonstrated that infectious viruses and bacteriophages can be synthesized in cell-free gene expression systems (42), and full genomic DNA can be replicated and mutated in a compartmentalized cell-free gene expression system (43).

We may indeed find a similar situation playing out as with whole-cell biosensors, where researchers either choose to focus their cell-free efforts on applications that appear more tractable from a regulatory perspective (which may not be the most necessary or beneficial applications), or they may find that their projects are ultimately unworkable from a funding or commercial standpoint.

## 7. Discussion

The challenges we have outlined through this paper strongly recall the Collingridge Dilemma whereby technologies at their early stages may be easier to control, when little is known about their applications and impacts (44). As these become apparent, governance becomes more difficult and expensive. Relatedly, the Pacing Problem describes situations where the rate of technological development far outstrips the agility and speed of governing institutions and systems (3). The dichotomy of deliberate release and contained use can be seen as an outcome of both of these phenomena: it made sense at the time it was conceived and enacted, based on the relatively few contemporary use cases of genetic engineering. However, as genetic engineering matured into synthetic biology, those product designations no longer hold. The regulations, or at least their implementation, have not kept pace and adapted to enable projects such as the arsenic biosensor discussed here to be implemented. On the other hand, regulations have flexed sufficiently to enable the use of synthetic biology products in new environments such as textile dye houses, albeit with significant transaction costs.

Some of the ways in which synthetic biologists have engaged with the dual challenges of control and of pacing involve embedding practices within the innovation process that extend beyond formal regulatory frameworks. Responsible Research and Innovation (45, 46) , for example, is a way of looking at technology governance that includes multi-stakeholder engagement, reflexivity and a deep consideration of values and motivations. The Arsenic Biosensor Collaboration, through its emphasis on stakeholder engagement and recognition of the cultural context for which it was being developed, might well be seen as an example of this. Recent engineered-insect projects, such as Target Malaria, make it explicit as one of their aims ‘We aim to achieve excellence in all areas of our work, creating a path for responsible research and development of genetic technologies, such as gene drive.’(47).

This framing is especially important for technologies where the regulatory situation is unclear. Future uses of cell-free synthetic biology will need to attend to dimensions of responsibility beyond solely what is ‘legal’, while they negotiate the inevitable uncertainties. This can be done through a variety of processes that implement responsible research and innovation , be that safety-by-design (22), midstream modulation (48) (or anticipatory governance (49).

However, these issues affect more than just the individual projects themselves, nor can they be ‘solved’ by individual teams of researchers and developers alone: they have far-reaching impacts and will require collaborative, imaginative and forward-thinking approaches that include stakeholders at multiple levels and in multiple geographies.

### 7.1 Regulation and competitiveness in the bioeconomy

The application of synthetic biology is predicted to yield important economic gains, a biotechnological industrial revolution in the form of powerful ‘bioeconomies’. The UK, for example, has been quite explicit about this, with a recent policy brief highlighting the field’s ‘game-changing (potential’ (50). In this reading, inappropriate or inefficient regulatory regimes are a barrier to entrepreneurship and national prosperity, a key fear being that nations whose regulations are overly stringent will ‘fall behind’ those with more lax regimes. Historically the EU has had one of the more rigid regimes. The failure of precautionary principle approaches to take into consideration potential benefits is a contributing factor to this lack of adaptability, which is illustrated by the lack of products entering the Annex II, Part C pathway that is intended to exempt products that meet strict biosafety criteria from the scope of the Directive as a whole.

A key pillar of the UK policy brief is the need to ‘Regulate & Reassure’ by taking advantage of its recent exit from the EU to ‘adapt its regulatory system to … open … up new markets for UK-based companies’(50). This conversation is being had: in the UK, the Regulatory Horizons Council—a body tasked with exploring post-Brexit regulatory opportunities in a number of sectors including genetic technologies—published a report recommending a move from process to product, noting that ‘the European regulatory system for genetic technologies is inhibiting useful innovation, disadvantaging farmers, and depriving us of useful future products that could help to meet societal needs’ (51). Gronvall, in her examination of US competitiveness, is more explicit about the economic impact, pointing to strict GMO regulations in the EU as especially detrimental. Gronvall warns of increasing anti-GMO feelings in the USA, voicing a fear that this, through ‘pressure placed on lawmakers to adhere to the precautionary principle’, would diminish ‘US competitiveness, particularly when it comes to realizing beneficial synthetic biology applications’ (52).

While national regulations matter, the level of alignment of regulation internationally is also economically important for trade reasons. Export controls of genetically modified products are complex, and GM products are making their way into markets with significant volumes of trade, adding complications (53).

### 7.2 Clarity and certainty are important for reaching the market

Certainty is a commercial benefit, and so, certainty that a particular area of biotechnology is well and sensibly regulated serves the growth of the field as a whole. This certainty need not be an assurance that a particular regulatory pathway is ‘easy’, but rather that it is ‘navigable and predictable’. That only a single company has managed to place a GM plant not related to food or feed on the market in the EU since 2001 is telling. Only one GMO has obtained authorization to be placed on the market in the EU: GM *Dianthus caryophyllus* carnations that are sold as cut flowers rather than whole plants and are marketed by the Australian company Florigen owned by Suntory Holdings Ltd, Osaka, Japan.

For the ‘market stage’ of EC/2001/18/EC, only seven plants and no other organisms have been approved for placing on the market. These are all carnations marketed by a single company or its subsidiaries and can only be imported to the EU in the form of cut flowers which cannot propagate. This lack of diversity points to the notion that navigating the regulatory system can be a more critical skill in biotechnology innovation than the technology itself, a ‘formula for success’ that, once figured out, can be repeated but not easily deviated from. A call for evidence in the UK’s House of Lords (35) sheds some light as to why this has been the case. First, it would appear that the procedure for plants itself is ‘failing lamentably’ and ‘not working as intended’ (35), largely, according to George Eustace MP, due to EU Member States’ ‘political objections’ (35) leading to an inability to achieve a qualified majority in subsequent votes.

Getting regulatory approval in the countries targeted by Colorifix was possibly facilitated because textile dyeing is an economically important industry where the chemical processes do serious environmental damage and alternative methods are actively being sought to reduce energy and water consumption and reduce polluting wastes. As a result, it is clear in the example of Colorifix’s regulatory approval in Bosnia that beneficial impact aligned with high-priority societal and political concerns in environmental but also economic terms could be a key driver of regulatory change.

### 7.3 European regulation and global impacts

European regulatory frameworks have had impacts far beyond the borders of the EU. Particularly in relation to GM plants, the ‘important influence the EU has in shaping regulatory policies in Africa due to trade relations and historical ties’ has been noted (54) (). This influence is more starkly conveyed by a scientist considering the regulatory situation in Honduras: ‘… industrialized countries also have a moral imperative not to influence policies that limit development of other less advanced countries and to learn from the missteps of regulating genetic engineering that illustrate that choosing a flawed paradigm has critical implications for a technology’ (55)).

The level of regulatory alignment and influence between the EU and LMICs is important because of growing interest in the potentially transformative development and use of synthetic biology products in LMICs beyond communities acting as ‘recipients’ of externally developed products such as biosensors or insects. This is in part thanks to the apparently reduced barrier to entry and cost-effectiveness of recent biological engineering tools. Techniques like CRISPR/Cas-gene editing are attractive in improved precision while reducing costs and time spent on lengthy manipulations. Synthetic biology could therefore be an appealing way to ‘solve local problems locally’ by harnessing in-country capacity. However, this will require the fostering of local regulatory regimes that are equipped to cope with novel biotechnologies being employed and establishing trust in these regimes. The shortcomings of GM-relevant regulatory frameworks in some LMICs have been discussed even without specific reference to synthetic biology: for example, they have been blamed for a relative lack of success in marketing GM crops and difficulties in establishing local bioeconomies (56). There are now a growing number of countries breaking alignment with EU regulation when it comes to the classification of gene-edited crops and this ongoing process has been extensively reviewed elsewhere(57, 58).

Several challenges are apparent here in both the frameworks themselves and their practical application. Regulatory capacity in the form of personnel is likely to be lacking, but traditional capacity building under the UN Environment Programme–Global Environment Facility’s program has been criticized as being both unsuitable (due to intransigent prescriptions) and unsustainable (e.g. focusing on one-off workshops). Additionally, it has been argued, ‘poor (*or no*) decision-making … continue[s] to undermine effective deployment of GMOs’ (emphasis added) (56). It is notable therefore that a lack of decision-making is something we also observe in the EU case studies described earlier. Indecisiveness in the political sphere is also highlighted as a major challenge in the establishment of effective biosafety regulatory systems in Africa (54), demonstrating again that interpretation and implementation of regulations are as important as the frameworks themselves.

### 7.4 Considering containment and release for future bioengineering

We conclude that the binary distinction between contained use and deliberate release, strongly embedded within the EU’s GMO regulation, is becoming less helpful over time. When it was put in place, the bifurcation of regulatory pathways was largely in step with the dominant GM technologies reaching the market. Now, as synthetic biology advances into new industrial sectors, transforms new organisms and begins to rewrite the rules of genetic transmission, the boundary between these two classifications is increasingly blurred. We have presented several examples of products or technologies that flex the definition of existing containment classifications, in some cases successfully. We also presented a whole-cell arsenic biosensor that failed to navigate the existing regulations and suggest this was partly because it was a clear example of a product that involved neither contained use nor deliberate release, as defined in the current EU Directives. Falling outside of these classifications has major practical and administrative implications because the mechanisms for approval and assessment at an EU level have been built around this split, including allocation of cases to different directorates and committees. In the case of the arsenic biosensor, this resulted in a lack of ownership and accountability by any regulatory body and ultimately ended in stasis and indecision.

We propose that the Asenic Biosensor Collaboration example could be more accurately characterized as undergoing ‘contained release’, due to its multiple layers of physical and genetic containment. Consequently, we suggest that containment should be considered as a wider spectrum of options. The current system forces product developers to choose between two paths, with a potential but effectively unused third route to exempt the product from the Directive. However, whichever path is chosen, many factors are in common during the in-depth GM risk assessments that are undertaken and which capture information on containment measures, including the increasingly important role of genetic containment. This points to the possibility of a more flexible regulatory pathway that tailors the necessary containment classification, measures and procedures to the specific risks of the product.

We recommend that future regulatory systems in the EU or elsewhere should have a unified point of entry and either a single pathway that considers all levels of containment or more pathways that anticipate the much broader range of products that emerge from biological engineering. To increase accountability for decision-making and decrease the risk of products being passed between multiple regulatory committees, there should be one or more regulatory bodies that are capable to review a full spectrum of containment options, with input from relevant specialist agencies. We have seen that the direction of travel of EU regulation influences that of other countries, particularly LMICs that are still building their own regulatory capacity. It is therefore even more imperative that the regulation of synthetic biology products in Europe creates an enabling, safe and responsible environment for the future bioeconomy by addressing the pathway to market for products that are in between or beyond the contained use and deliberate release divide.
